# The distribution of refraction by age and gender in a non-myopic Chinese children population aged 6–12 years

**DOI:** 10.1186/s12886-020-01709-1

**Published:** 2020-11-07

**Authors:** Xiyan Zhang, Yonglin Zhou, Jie Yang, Yan Wang, Wenyi Yang, Liuwei Gao, Yao Xiang, Fengyun Zhang

**Affiliations:** 1 Department of Child and Adolescent Health Promotion, Jiangsu Provincial Center for Disease Control and Prevention, Nanjing, China; 2 Public Health Research Institute of Jiangsu Province, Nanjing, China; 3 School of Public Health, Southeast University, Nanjing, Jiangsu Province China; 4 Current Address: No.172 Jiangsu Road, Nanjing, 210009 China

**Keywords:** Non-myopia, Distribution of refraction, Children, Alarming threshold values, Jiangsu province

## Abstract

**Background:**

The Prevalence of myopia is increasing in China. This study aimed to explore the distribution of spherical equivalent (SE) and its association with age, body mass index (BMI), gender in a non-myopic Chinese children population aged 6 to 12 years.

**Methods:**

A total of 6362 students were recruited for ophthalmological investigation. Demographic and myopia related behavioral information was collected. SE value was measured by the Topcon RM-8900 or KR-800autorefractors. Potential independent risk factors were determined with Odds Ratio (OR) and 95% Confidence Interval (CI) by logistic regression analysis. We further constructed the nomogram model to predict future onset of myopia.

**Results:**

Among the study population, 3900 (61.3%) were non-myopic. The prevalence of myopia is 38.0% for boys and 39.5% for girls. The average SE values were 0.50 ± 0.70 D for boys and 0.60 ± 0.80 D for girls. The mean SE values decreased with age, and the value of height and BMI took on a stable trend. Threshold values for myopia varied across age groups and gender. Paternal myopia (OR: 1.22, 95%CI: 1.01–1.48), near-work activities on weekends (2.56, 1.17–5.61), and outdoor activities (0.68, 0.54–0.86) were associated with potential myopic in students.

**Conclusion:**

A series of age-gender based SE threshold values were established to predict myopia in Chinese children aged 6 to 12 years. High risk factors for myopia included paternal myopia, near-work activities on weekends, and outdoor activities. Countermeasures are encouraged to reverse the increasing trend of myopia in children.

**Supplementary Information:**

The online version contains supplementary material available at 10.1186/s12886-020-01709-1.

## Background

There is a dramatic increase in the prevalence of myopia in Eastern Asia. In China, a great increase was also seen in the young generation, indicating the importance of prediction of early-onset myopia among juveniles [1–3]. When it comes to the intervention of myopia, people often pay more attention to treatment such as optical or pharmaceutical methods to slow down eye growth, and thus retard the progression of myopia [4]. However, from a public health perspective, it is more desirable for non-myopic students to develop an early warning comprehensive system to predict and prevent the onset of myopia.

Holden et al. stated the future development trends of myopia from a macroscopic view, predicting that nearly half of the world’s population may be myopic by 2050, with as much as 10% having highly myopic [5]. However, the prediction of myopia on an individual level is urgent and relevant research is limited. Karla Zadnik et al. noted that spherical equivalent (SE) refractive error is the best single predictor of future myopia, comparing to other factors such as parental myopia, near work, and outdoor activities [6]. In Beijing three-year follow-up eye study, researchers found that children aged 6 to 7 years showed a significant SE decrease, AL increase, CCT thickening, ACD deepen, LT thinning, and AL/CR increase. These findings may be an early warning signal of myopia development [7]. BMI is a reliable indicator of risk, growth, and childhood related disease such as obesity, elevated blood pressure, and so on [8, 9]. Obese children aged 7 to 9 years would be more likely to have poor visions comparing to those without obesity, and such influence could last to 12 years of age among boys [10]. A nomogram or nomograph is a form of line chart showing scales for all variables involved in a formula, it is a rule functioned as a simple calculator [11]. Currently, there is a growing application of the nomogram model using rational risk factors in predicting the probability of occurrence, progression/prognosis of an individual’s disease [12–14]. Therefore, it is essential to predict the myopia onset based on factors such as sex, age, BMI, and SE.

This study aimed to explore characteristics of the distribution of refraction, including age, BMI, sex, and SE value, among non-myopic Chinese children. Alarming threshold values were proposed to predict future myopia, and associations between alarming threshold values and factors associated with myopia were addressed.

## Methods

### Study sites and populations

This study was based on the program “Surveillance for common disease and health risk factors among students, sub-program: ophthalmological investigation” during the 2018–2019 academic year in Jiangsu Province. We enrolled 26,461 students aged 6–17 years from 12 regions in Jiangsu Province (Supplement figure 1) in this program, and A total of 6363 primary school students were aged from 6 to 12.5, participated in the sub-program: ophthalmological physical examination.

The inclusion criteria for our subjects were (1) non-myopic children and lack of other serious eye diseases;(2) Chinese Han Nationality Students;(3) age between 6 to 12;(4) Ability of parents/guardians to provide informed consent.

### Data collection and ethics statement

Myopia was defined as − 0.50 diopters(D) in the worse eye and the worse eye was defined as the eye with the greater absolute value of refractive error (spherical equivalent). All students took part in this sub-program, and they were required to provide basic demographic information including name, sex, regional, and some of their parents filled in questionnaires concerning myopic related questions. Detailed information could be listed as follows: Electronic questionnaire was used in this study. The child’s parents filled the form by logging into wechat app. The establishment of the questionnaire was based on the The SCORM cohort study [15, 16]. It would take parents 30-40 min to finish this questionnaire. The main contents include the following three aspects. Firstly, basic information including parental myopia and Whether the child had brothers/sisters. Secondly, learning-related issues including the near work activities. Thirdly, work and rest related issues including outdoor activities, the duration of sleep.

An autorefractor (Topcon RM-8900 or KR-800; Topcon Co., Tokyo, Japan) was applied with cycloplegia. The cycloplegic refraction is measured using tropicamide phenylephrine eye drops every 5 min,3 times, and then the refractive error is measured 30 min after the first drop of tropicamide by autorefractor with five repeated measurements. The spherical equivalent of the refractive error was calculated as the spherical value of refractive error plus one half of the cylindrical value.

The study protocol was approved by the Institutional Review Board of Ethics committee of Jiangsu Province, and detailed information can be found in the previous article [10]. We used an autorefractor with cycloplegia under parents’ informed consent.

### Statistical analysis

The age-BMI-specific spherical equivalent values were calculated for the percentiles of 5th, 10th, 25th,50th,75th,90th, 95th for both boys and girls. We used 5th, 10th, 25th,50th,75th,90th and 95th percentiles spherical equivalent values as potential age-specific alert spherical equivalent values. (Supplement Table 2) According to previous studies [17], Jiangsu Province had the top prevalence of reduced visual acuity (76.2%), and then we set the upper limit value (UPPER) for the non-myopic population as 76.2%. Then the alarming threshold value percentile for non-myopic students can be calculated as follows:

$$ Alert\kern0.17em Value\kern0.17em Percentiles=\frac{\mathrm{UPPER}(0.762)\;\mathrm{X}\;\mathrm{Total}\kern0.17em \mathrm{Number}(6362)\hbox{-} \mathrm{Number}\kern0.17em \mathrm{of}\kern0.17em \mathrm{myopic}\kern0.17em \mathrm{students}\;(2462)}{\mathrm{Number}\kern0.17em \mathrm{of}\kern0.17em \mathrm{non}\hbox{-} \mathrm{myopic}\kern0.17em \mathrm{students}\;(3900)} $$

Therefore, we selected 50th spherical equivalent values as alert values based on the calculated *Alert Value Percentiles*.

Then we verified the correctness of the alarming threshold value by calculating OR values with proven myopia related factors, such as outdoor activity time and parental myopia.

We then performed a logistic model to select variables fit for the nomogram model. Nomograph was drawn by R software with rms packages. Statistical analyses were performed with R software (www.R-project. Org, version 3.5.3) with additional rms package [18] Detailed calculation principle can be found in previous studies [19]. Besides, continuous variables were presented as the mean with standard deviation (SD).

## Results

### Baseline characteristics of this study

The prevalence of myopia for children aged 6 to 12 was 38.0% for boys and 39.5% for girls. We eventually obtained 3900 non-myopic students, and the ratio of male to female was 1.16. The mean SE decreased with age in children, and the value of height and BMI took on a stable trend. (Table 1) Also, among 6362 students we found that myopic boys or girls had higher values of BMI or height than these of non-myopic boys or girls (P<0.001). (Supplement Table 1).

**Table 1:** Characteristics of non-myopic students from Jiangsu Province

Age	Numbers of non-myopic students		Prevalence of myopia, %		SE, D		Height, cm		BMI, kg/m2	
	Boys	Boys	Boys	Girls	Boys	Girls	Boys	Girls	Boys	Girls
6	263	232	9.3	4.9	0.60 ± 0.80	0.90 ± 0.90	122.0 ± 6.1	119.5 ± 5.7	17.4 ± 3.0	16.3 ± 2.1
6.5	127	123	15.3	8.2	0.70 ± 0.70	0.70 ± 0.70	124.1 ± 6.9	123.7 ± 7.4	17.4 ± 2.8	16.6 ± 2.0
7	286	206	17.1	15.9	0.70 ± 0.80	0.50 ± 0.60	126.8 ± 5.6	124.9 ± 5.1	17.4 ± 2.7	16.8 ± 2.7
7.5	259	268	15.4	16.5	0.70 ± 0.70	0.80 ± 1.00	125.6 ± 6.4	124.7 ± 7.2	17.4 ± 2.9	16.6 ± 2.4
8	227	218	29.7	26.6	0.50 ± 0.90	0.50 ± 0.80	133.2 ± 6.2	132.4 ± 6.5	17.9 ± 3.2	17.0 ± 2.8
8.5	232	192	22.1	25.6	0.50 ± 0.60	0.60 ± 1.00	132.0 ± 6.4	129.2 ± 6.2	18.2 ± 3.3	17.3 ± 2.7
9	98	86	44.6	49.4	0.50 ± 0.70	0.50 ± 0.80	138.0 ± 6.8	138.0 ± 6.3	18.9 ± 3.5	17.9 ± 3.6
9.5	196	174	35.9	38.5	0.50 ± 0.70	0.50 ± 0.80	136.5 ± 6.6	135.5 ± 6.8	18.5 ± 3.5	17.5 ± 2.9
10	87	88	60.8	53.2	0.30 ± 0.50	0.40 ± 0.70	143.5 ± 6.7	143.8 ± 7.4	19.9 ± 4.3	18.6 ± 3.5
10.5	105	71	52.1	61.4	0.40 ± 0.60	0.40 ± 0.70	142.9 ± 7.9	143.1 ± 6.8	19.4 ± 3.6	18.2 ± 3.4
11	60	24	68.8	82.1	0.40 ± 0.70	0.30 ± 0.60	148.8 ± 8.2	151.5 ± 8.5	20.8 ± 5.0	19.6 ± 4.3
11.5	65	52	67.5	74.1	0.40 ± 0.80	0.50 ± 0.90	149.9 ± 9.5	148.8 ± 6.5	21.5 ± 4.4	18.9 ± 3.1
12	43	35	74.7	78.0	0.30 ± 0.50	0.30 ± 0.80	153.6 ± 8.1	153.6 ± 6.3	19.8 ± 3.8	19.8 ± 3.3
12.5	48	35	73.8	78.7	0.20 ± 1.50	0.20 ± 0.50	156.0 ± 9.2	154.6 ± 7.2	20.3 ± 3.4	19.7 ± 3.8
Total	2096	1804	38.0	39.5	0.50 ± 0.70	0.60 ± 0.80	132.7 ± 11.2	130.9 ± 11.3	18.3 ± 3.5	17.2 ± 2.9

### Alert values for non-myopic students according to percentiles of BMI and SE

**Fig. 1:**
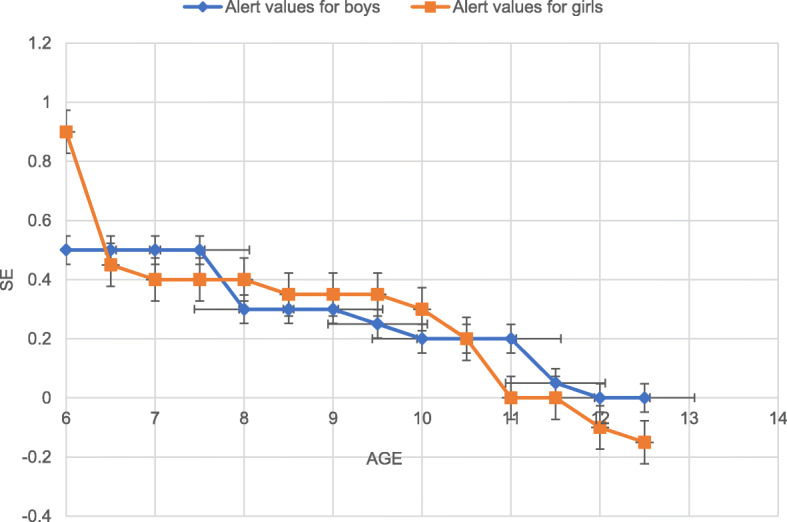
Distribution of refraction by age and gender in a non-myopic Chinese children population aged 6–12 years (50th percentiles)

The supplement Table 2 showed age-BMI-specific spherical equivalent values of 5th, 10th, 25th,50th,75th,90th and 95th for both sexes. We set 50th percentiles cut-off points as: for children aged 6 years of age, 0.40–0.60 D for boys and 0.80–1.00 D for girls respectively. For children aged 7 years, 0.40–0.60 D for boys and 0.30–0.50 D for girls respectively, For children aged 8 years, 0.20–0.40 D for boys and 0.30–0.50D for girls, For children aged 9 years, 0.20–0.40 D for boys and 0.30–0.40D for girls, 0.10–0.30 D for boys aged 10 and 0.30D for girls aged 10 years, 0.10–0.30 D for boys aged 11 and − 0.30-0.30 D for girls aged 11 years, and for children aged 12, − 0.10-0.10 D for boys and − 0.30-0.10 D for girls aged 12 years. (Fig. 1).

### Relationship between myopic alarming threshold for non-myopic students and myopia associated factors

**Fig. 2:**
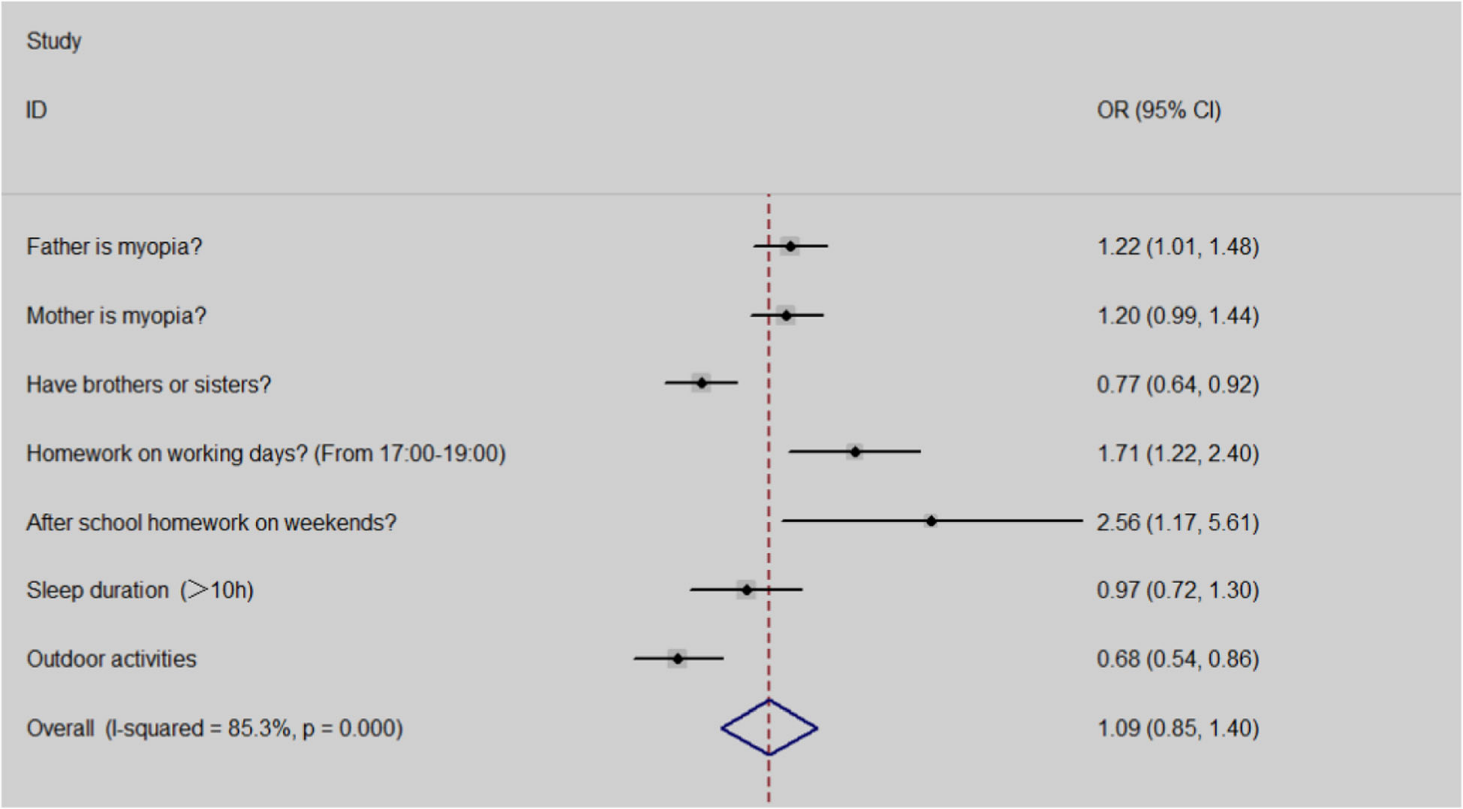
Forest graph of relationship between alert values for non-myopic students and myopic factors

Non-myopic students with alarming threshold had a higher proportion of their myopic father. (P<0.05) A family that had more than one kid is a protective factor for non-myopic students to prevent the onset of myopia. After school homework especially on weekends had higher OR values for students with no myopia. (OR: OR:2.56,95%CI:1.17–5.61). Sleep and outdoor activities also had more impact on non-myopic students with alarming threshold. (Table 2, Fig. 2).

**Table 2:** Relationship between myopic alertion for non-myopic students and myopia associated factors

	Non-myopic students with no-alertion (%/mean ± SD)	non-myopic students with alertion (%/mean ± SD)	P
Father is myopia?	31.5 (404/1284)	35.9 (260/725)	0.048
Mother is myopia?	34.7 (446/1284)	38.9 (282/725)	0.062
Have brothers or sisters?	42.9 (551/1284)	36.6 (265/725)	0.005
After school homework on working days? (From 17:00–19:00)	34.1 (138/405)	46.9 (98/209)	0.002
After school homework on weekends? (From 20:00–22:00)	1.0 (11/1098)	2.5 (15/594)	0.015
Sleep duration on working days	9.68 ± 0.75	9.60 ± 0.82	0.004
Outdoor activities on weekends?	84.2 (1081/1284)	78.3 (568/725)	0.001

### Nomograph for predicting childhood myopia onset

**Fig. 3:**
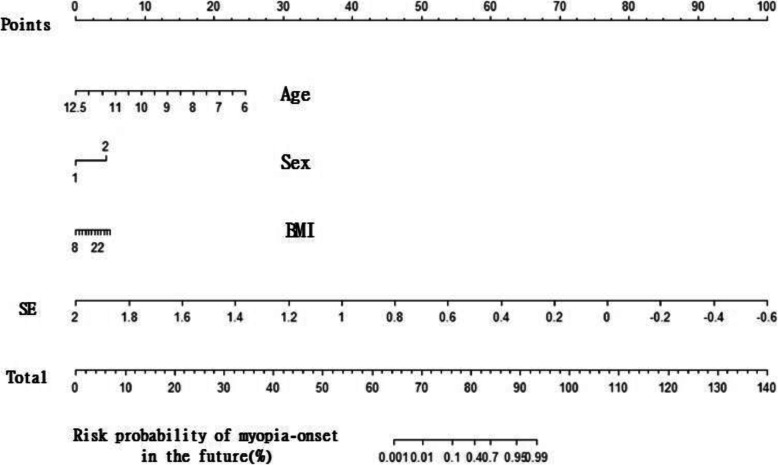
Nomogram for predicting childhood myopia onset

The nomogram graph showed that total points ranged from 64 to 93 corresponding to risk the probability of future myopia onset in half-year ranged from 0 to 100%. When SE values ranged from − 0.60 to 2.00, the corresponding points ranged from 100 to 0. Observation’s age increased (from 6 to 12.5) with the corresponding points decreased (25 to 0). Sex seemed to have little impact on future myopia onset: male and female are corresponding to 0 and 4 points respectively. The relationship between BMI and points can be presented as 8–0 points, 12–1 points, 16–2 points, 20–3 points, 24–4 points, and 30–5 points. (Fig. 3).

## Discussion

This study mainly aimed to present the distribution of refraction in a non-myopic Chinese children population aged 6–12 years and tried to develop an alarming threshold for predicting the future onset of myopia. The sample of the study is quite big and it is of great value in guiding the practice of prevention and control of myopia in the public health field. This is the first study using age, BMI, and sex to propose alert values and predict myopia onset among children aged 6 to 12 years in the world. It is also the first time using a nomogram model to predict the risk of myopia onset among children. Moreover, we addressed the epidemiology characteristics for non-myopic Chinese children aged 6 to 12 years in Jiangsu Province, China for the first time.

The prevalence of myopia among children aged 6 to 12 years was 38.0% for boys and 39.5% for girls, which were higher than Chinese adults. The prevalence of myopia for definitions of SE of <− 0.50 was reported to be 22.9% (95%CI: 21.7–24.2) in the Beijing Eye Study (*n* = 4439, aged 40–90 years) [20]. Both values of height and BMI for myopic children were higher than those without myopia.

Hirsch et al. firstly noted that the refractive error had the ability to predict later myopia onset (children aged 6 years) [21]. Thirty years later, Zadniket et al. developed the first model to predict the onset of myopia among children [22]. Karla Zadnik et al. concluded that SE refractive error was the single best predictor [6] and set cut-off points as + 0.75 D for children aged 6 years, + 0.50 D for children aged 7 to 8 years, + 0.25 D for children aged 9 to 10 years, and + 0.00 D for children aged 11 years. The trend of cut-off points is similar to our alert values, but the detailed information might be different. Larger datasets and longer follow-up are needed to better predict the cut-off points.

Heredity, outdoor activities, and near work had a profound influence on the onset and progression of myopia [23]. In this study, these factors also had a significant impact on non-myopic children. Nomograms may be valuable tools to estimate the likelihood of diseases in the future [18]. Based on alert values, we built up a nomogram model to give warnings to children with alarming threshold.

There were some limitations to this study. Firstly, cut-off points were built based on a cross-sectional study, and the small sample size in the high age groups could have an influence on the distribution of refraction. For students with high age especially older than 11 most of them are nearsighted. Therefore, a long-term cohort study is needed to improve the accuracy of alarming threshold values. Secondly, higher sensitive factors associated with myopia required further analysis for better forecasting. As we all know that major environmental risk factors including extended near work and minimal outdoor exposure had a greater impact on the onset of myopia [24, 25], and continuous data obtained in the form of non-questionnaire form may be more sensitive. Thirdly, two types of Topcon refractors (RM-8900 or KR-800) were used in the study, and the precision of two types might affect the accuracy of our study. Last but not least, we choose tropicamide according to National Guidelines for the Prevention and Treatment of Myopia [26] considering the large population in this survey, the duration of tropicamide eye drops is short, and the action intensity is weak. Using tropicamide rather than cyclopentolate eye drops for cycloplegic refraction might have an impact on the results.

## Conclusion

This study presented the distribution of refraction for non-myopic students in Jiangsu Province, China. A series of alarming threshold values were proposed to provide early warning reference for Chinese children aged 6 to 12 years. Heredity, near work, and outdoor activities had an impact on non-myopic students with myopic alarming threshold, and sensitive continuous data concerning risk factors mentioned above should be explored to be used as an early alert value in the future.

## Supplementary Information

**Additional file 1.**

## Data Availability

The datasets generated and/or analysed during the current study are not publicly available because of involving students’ personal privacy, but are available from the corresponding author on reasonable request.

## Abbreviations

SE: Spherical equivalent
BMI: Body mass index

## References

[1] He M, Zheng YF (2009). Prevalence of myopia in urban and rural children in mainland China. Optometry Vision Sci.

[2] Mingzhi Z, Liping L, Lizhen C (2010). Population density and refractive error among Chinese children. Invest Ophthalmol Vis Sci.

[3] Bo LY, Tien Yin W, Ping SL (2009). Refractive errors in a rural Chinese adult population the Handan eye study. Ophthalmology.

[4] Huang J, Wen D, Wang Q (2016). Efficacy comparison of 16 interventions for myopia control in children: a network meta-analysis. Ophthalmology.

[5] Holden BA, Fricke TR, Wilson DA (2016). Global prevalence of myopia and high myopia and temporal trends from 2000 through 2050. OPHTHALMOLOGY.

[6] Zadnik K, Sinnott LT, Cotter SA (2015). Prediction of juvenile-onset myopia. JAMA Ophthalmol.

[7] Min WU, Ning-Dong LI, Guo Y, et al. Three-year cohort study on the changes of ocular biological parameters of school children. Ophthalmol Chin. 2016;2016(02).

[8] Freedman DS, Sherry B (2009). The validity of BMI as an indicator of body fatness and risk among children. Pediatrics.

[9] Nyamdorj R, Qiao Q, Group DS (2012). BMI compared with central obesity indicators in relation to diabetes and hypertension in Asians. Obesity.

[10] Zhang X, Zhang F, Yang J. Prevalence of overweight and obesity among primary school-aged children in Jiangsu Province, China, 2014-2017. PLoS One. 2018;13(8):e0202681. 10.1371/journal.pone.0202681 PMC6107224 30138424

[11] Grimes DA (2008). The nomogram epidemic: resurgence of a medical relic. Ann Intern Med.

[12] Shariat SF, Kattan MW, Vickers AJ (2009). Critical review of prostate cancer predictive tools. Future Oncol.

[13] Rochester MA, Pashayan N, Matthews F (2009). Development and validation of risk score for predicting positive repeat prostate biopsy in patients with a previous negative biopsy in a UK population. BMC Urol.

[14] Mazouni C, Spyratos F, Romain S (2012). A nomogram to predict individual prognosis in node-negative breast carcinoma. Eur J Cancer.

[15] Dirani M, Tong L, Gazzard G (2009). Outdoor activity and myopia in Singapore teenage children. Brit J Ophthalmol.

[16] Seang-Mei S, Anoop S, Tan SB (2006). A cohort study of incident myopia in Singaporean children. Invest Ophth Vis Sci.

[17] Song Y, Hu P, Dong Y (2017). Prealence of reduced visual acuity among Chinese Han students in 2014. J Peking Univ (Health Sciences).

[18] Harrell JF: Rms: regression modeling strategies. R package version 4.3–1. 2013.

[19] Iasonos A, Schrag D, Raj GV (2008). How to build and interpret a Nomogram for Cancer prognosis. J Clin Oncol.

[20] PAN CW, Ramamurthy D, Saw SM (2011). Worldwide prevalence and risk factors for myopia. Ophthalmic Physiol Optics.

[21] Hirsch MJ (1964). Predictability of refraction at age 14 on the basis of testing at age 6--interim report from the OJAI longitudinal study of refraction. Am J Optometry Archiv Am Acad Optometry.

[22] Zadnik K, Mutti DO, Friedman NE (1999). Ocular predictors of the onset of juvenile myopia. Invest Ophthalmol Vis Sci.

[23] Mutti DO, Mitchell GL, Moeschberger ML (2002). Parental myopia, near work, school achievement, and children's refractive error. Invest Ophthalmol Vis Sci.

[24] Tideman JWL, Polling JR, Hofman A, et al: Environmental factors explain socioeconomic prevalence differences in myopia in 6-year-old children. Brit J Ophthalmol. 2017;bjophthalmol-2017-310292. 10.1136/bjophthalmol-2017-310292 28607175

[25] Huang HM, Chang ST, Wu PC. The association between near work activities and myopia in children&mdash;a systematic review and meta-analysis. PLoS One. 2015;10:e140419. 10.1371/journal.pone.0140419 PMC4618477 26485393

[26] National Guidelines for the Prevention and Treatment of Myopia. http://www.nhc.gov.cn/yzygj/s7652/201806/41974899de984947b8faef92a15e9172.shtml.

