# Supplementary material for: The distribution of refraction by age and gender in a non-myopic Chinese children population aged 6–12 years
Source: BMC Ophthalmol. 2020 Nov 7;20:439. doi: 10.1186/s12886-020-01709-1 (PMC7648976; doi:10.1186/s12886-020-01709-1)
Supplement: Supplementary file 1 — Additional file 1. [file 12886_2020_1709_MOESM1_ESM.docx]

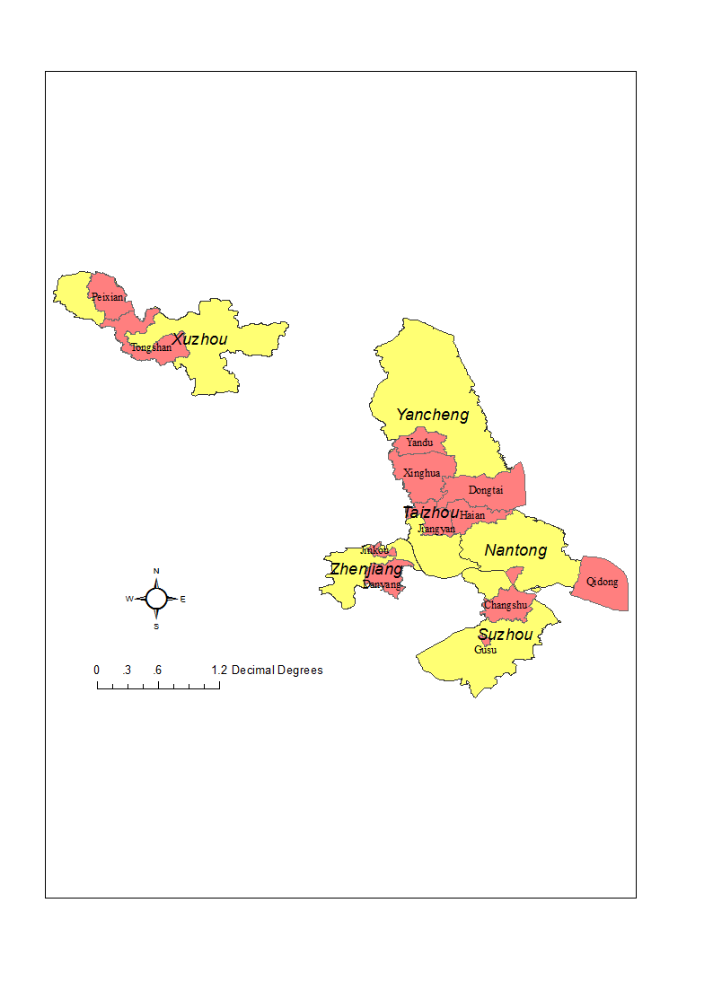


Supplement figure1 Region distribution of primary school students from Jiangsu Province Eye Study

* Supplementary Figure 1 my own

Supplement table1 Comparing between myopic and non-myopic students from Jiangsu Province

|  | | Mean | F | P | T | df |
| --- | --- | --- | --- | --- | --- | --- |
|  |  |  |  |  |  |  |
| Height | myopic boys | 144.12 | 24.56 | 0.000 | 27.77 | 3380.00 |
|  | non-myopic boys | 132.66 |  |  | 27.09 | 2505.28 |
|  | myopic girls | 144.67 | 21.16 | 0.000 | 31.73 | 2980.00 |
|  | non-myopic girls | 130.91 |  |  | 31.31 | 2400.78 |
| BMI | myopic boys | 19.63 | 40.59 | 0.000 | 10.54 | 3380.00 |
|  | non-myopic boys | 18.26 |  |  | 10.22 | 2446.48 |
|  | myopic girls | 18.51 | 44.83 | 0.000 | 10.72 | 2980.00 |
|  | non-myopic girls | 17.24 |  |  | 10.33 | 2201.80 |

Supplement Table2 Percentiles of spherical equivalent value for boys and girls by age and height for primary students from Jiangsu Province Eye study

| Age | SE value | BMI percentiles for boys | | | | BMI percentiles for girls | | | |
| --- | --- | --- | --- | --- | --- | --- | --- | --- | --- |
|  |  | ＜25^th^ | ≥25^th^ | ≥50^th^ | ≥75^th^ | ＜25^th^ | ≥25^th^ | ≥50^th^ | ≥75^th^ |
| 6 | 95^th^ | -0.20 | -0.20 | -0.10 | 0.00 | -0.20 | -0.20 | 0.00 | 0.00 |
|  | 90^th^ | 0.00 | 0.00 | 0.00 | 0.00 | 0.00 | -0.10 | 0.00 | 0.00 |
|  | 75^th^ | 0.00 | 0.00 | 0.00 | +0.30 | +0.10 | +0.10 | +0.50 | +0.20 |
|  | 50^th^ | +0.40 | +0.40 | +0.40 | +0.60 | +0.80 | +0.90 | +1.00 | +0.80 |
|  | 25^th^ | +1.10 | +1.00 | +0.90 | +1.10 | +1.40 | +1.30 | +1.50 | +1.30 |
|  | 10^th^ | +1.80 | +1.50 | +1.20 | +1.60 | +1.90 | +1.80 | +1.90 | +1.50 |
|  | 5^th^ | +2.30 | +1.70 | +1.60 | +2.00 | +2.70 | +2.00 | +2.60 | +1.80 |
| 6.5 | 95^th^ | -0.20 | -0.20 | -0.20 | -0.30 | -0.20 | -0.20 | 0.00 | -0.20 |
|  | 90^th^ | 0.00 | -0.10 | -0.10 | -0.20 | 0.00 | -0.10 | 0.00 | 0.00 |
|  | 75^th^ | +0.10 | 0.00 | -0.10 | +0.30 | 0.00 | +0.10 | +0.20 | +0.20 |
|  | 50^th^ | +0.40 | +0.40 | +0.40 | +0.60 | +0.40 | +0.40 | +0.50 | +0.50 |
|  | 25^th^ | +1.10 | +0.90 | +0.90 | +1.00 | +1.10 | +1.20 | +1.20 | +0.90 |
|  | 10^th^ | +1.50 | +1.40 | +1.10 | +1.40 | +1.50 | +1.70 | +1.90 | +1.50 |
|  | 5^th^ | +1.50 | +1.50 | +1.30 | +1.80 | +1.90 | +1.80 | +2.00 | +1.60 |
| 7 | 95^th^ | -0.20 | -0.30 | -0.40 | -0.30 | -0.20 | -0.20 | -0.40 | -0.30 |
|  | 90^th^ | 0.00 | -0.10 | -0.20 | -0.20 | 0.00 | -0.10 | -0.30 | -0.20 |
|  | 75^th^ | +0.10 | 0.00 | -0.10 | 0.00 | 0.00 | 0.00 | 0.00 | +0.10 |
|  | 50^th^ | +0.40 | +0.40 | +0.40 | +0.60 | +0.30 | +0.40 | +0.50 | +0.50 |
|  | 25^th^ | +1.00 | +0.90 | +0.90 | +1.00 | +0.90 | +0.80 | +1.00 | +0.90 |
|  | 10^th^ | +1.20 | +1.40 | +1.10 | +1.50 | +1.10 | +1.20 | +1.20 | +1.50 |
|  | 5^th^ | +1.50 | +1.50 | +1.30 | +1.80 | +1.70 | +1.40 | +1.40 | +1.60 |
| 7.5 | 95^th^ | -0.20 | -0.30 | -0.40 | -0.30 | -0.30 | -0.20 | -0.40 | -0.30 |
|  | 90^th^ | 0.00 | -0.10 | -0.20 | -0.20 | -0.10 | -0.10 | -0.30 | -0.20 |
|  | 75^th^ | +0.10 | +0.00 | -0.10 | +0.00 | +0.00 | 0.00 | 0.00 | +0.10 |
|  | 50^th^ | +0.40 | +0.40 | +0.40 | +0.60 | +0.30 | +0.40 | +0.50 | +0.50 |
|  | 25^th^ | +0.90 | +0.90 | +0.90 | +1.00 | +0.90 | +0.80 | +1.00 | +0.90 |
|  | 10^th^ | +1.20 | +1.40 | +1.10 | +1.50 | +1.10 | +1.20 | +1.20 | +1.50 |
|  | 5^th^ | +1.50 | +1.50 | +1.30 | +1.60 | +1.70 | +1.40 | +1.40 | +1.60 |
| 8 | 95^th^ | -0.30 | -0.20 | -0.40 | -0.40 | -0.30 | -0.30 | -0.40 | -0.30 |
|  | 90^th^ | -0.30 | -0.10 | -0.30 | -0.20 | -0.10 | -0.10 | -0.30 | -0.20 |
|  | 75^th^ | 0.00 | 0.00 | -0.10 | +0.00 | 0.00 | 0.00 | 0.00 | +0.10 |
|  | 50^th^ | +0.30 | +0.40 | +0.30 | +0.20 | +0.30 | +0.40 | +0.40 | +0.50 |
|  | 25^th^ | +0.60 | +0.80 | +0.80 | +0.80 | +0.90 | +0.80 | +0.80 | +0.90 |
|  | 10^th^ | +1.00 | +1.30 | +1.10 | +1.30 | +1.10 | +1.20 | +1.20 | +1.50 |
|  | 5^th^ | +1.40 | +1.50 | +1.30 | +1.60 | +1.50 | +1.40 | +1.40 | +1.60 |
| 8.5 | 95^th^ | -0.30 | -0.30 | -0.40 | -0.40 | -0.30 | -0.30 | -0.40 | -0.30 |
|  | 90^th^ | -0.30 | -0.10 | -0.30 | -0.20 | -0.10 | -0.10 | -0.30 | -0.20 |
|  | 75^th^ | 0.00 | 0.00 | -0.10 | 0.00 | 0.00 | 0.00 | 0.00 | 0.00 |
|  | 50^th^ | +0.30 | +0.40 | +0.30 | +0.20 | +0.30 | +0.40 | +0.40 | +0.50 |
|  | 25^th^ | +0.60 | +0.80 | +0.80 | +0.80 | +0.90 | +0.80 | +0.80 | +0.90 |
|  | 10^th^ | +1.00 | +1.00 | +1.00 | +1.30 | +1.10 | +1.20 | +1.10 | +1.40 |
|  | 5^th^ | +1.40 | +1.30 | +1.30 | +1.60 | +1.50 | +1.40 | +1.40 | +1.60 |
| 9 | 95^th^ | -0.30 | -0.30 | -0.40 | -0.40 | -0.40 | -0.40 | -0.40 | -0.40 |
|  | 90^th^ | -0.30 | -0.10 | -0.30 | -0.20 | -0.30 | -0.30 | -0.30 | -0.30 |
|  | 75^th^ | 0.00 | 0.00 | +0.10 | 0.00 | -0.10 | 0.00 | 0.00 | 0.00 |
|  | 50^th^ | +0.30 | +0.40 | +0.30 | +0.20 | +0.30 | +0.30 | +0.40 | +0.40 |
|  | 25^th^ | +0.60 | +0.80 | +1.00 | +0.60 | +0.40 | +0.70 | +0.80 | +0.90 |
|  | 10^th^ | +1.00 | +1.00 | +1.60 | +1.00 | +0.80 | +1.20 | +1.10 | +1.40 |
|  | 5^th^ | +1.10 | +1.30 | +2.80 | +1.10 | +0.90 | +1.30 | +1.40 | +1.60 |
| 9.5 | 95^th^ | -0.30 | -0.40 | -0.40 | -0.40 | -0.40 | -0.40 | -0.40 | -0.40 |
|  | 90^th^ | -0.30 | -0.30 | -0.30 | -0.20 | -0.30 | -0.30 | -0.30 | -0.30 |
|  | 75^th^ | +0.00 | +0.00 | +0.00 | 0.00 | -0.10 | 0.00 | 0.00 | 0.00 |
|  | 50^th^ | +0.30 | +0.30 | +0.30 | +0.20 | +0.30 | +0.30 | +0.40 | +0.40 |
|  | 25^th^ | +0.60 | +0.60 | +0.90 | +0.60 | +0.40 | +0.70 | +0.80 | +0.90 |
|  | 10^th^ | +1.00 | +1.00 | +1.20 | +0.90 | +0.80 | +1.10 | +1.10 | +1.20 |
|  | 5^th^ | +1.10 | +1.20 | +1.60 | +1.00 | +0.90 | +1.30 | +1.40 | +1.40 |
| 10 | 95^th^ | -0.40 | -0.40 | -0.40 | -0.40 | -0.40 | -0.40 | -0.40 | -0.40 |
|  | 90^th^ | -0.40 | -0.40 | -0.10 | -0.20 | -0.30 | -0.30 | -0.30 | -0.40 |
|  | 75^th^ | -0.30 | 0.00 | 0.00 | 0.00 | -0.10 | -0.20 | -0.20 | -0.20 |
|  | 50^th^ | +0.10 | +0.30 | +0.30 | +0.20 | +0.30 | +0.30 | +0.30 | +0.30 |
|  | 25^th^ | +0.60 | +0.60 | +0.50 | +0.60 | +0.40 | +0.60 | +0.40 | +0.50 |
|  | 10^th^ | +0.90 | +0.80 | +0.60 | +0.90 | +0.80 | +1.10 | +1.00 | +1.00 |
|  | 5^th^ | +0.90 | +0.90 | +0.80 | +1.00 | +0.90 | +1.30 | +1.40 | +1.40 |
| 10.5 | 95^th^ | -0.10 | -0.40 | -0.40 | -0.40 | -0.40 | -0.40 | -0.40 | -0.40 |
|  | 90^th^ | -0.10 | -0.30 | -0.10 | -0.40 | -0.40 | -0.30 | -0.30 | -0.40 |
|  | 75^th^ | -0.30 | -0.10 | 0.00 | -0.30 | -0.10 | -0.20 | -0.20 | -0.20 |
|  | 50^th^ | +0.10 | +0.10 | +0.30 | +0.20 | +0.30 | +0.30 | +0.30 | +0.10 |
|  | 25^th^ | +0.80 | +0.50 | +0.50 | +0.60 | +0.40 | +0.60 | +0.40 | +0.50 |
|  | 10^th^ | +0.90 | +0.80 | +0.60 | +0.90 | +0.80 | +1.10 | +1.00 | +1.00 |
|  | 5^th^ | +0.90 | +0.90 | +0.80 | +1.00 | +0.90 | +1.30 | +1.40 | +1.40 |
| 11.0 | 95^th^ | -0.30 | -0.40 | -0.40 | -0.40 | -0.40 | -0.40 | -0.40 | -0.40 |
|  | 90^th^ | -0.20 | -0.40 | -0.10 | -0.40 | -0.40 | -0.40 | -0.30 | -0.40 |
|  | 75^th^ | -0.30 | -0.30 | 0.00 | -0.30 | -0.30 | -0.30 | -0.20 | -0.20 |
|  | 50^th^ | +0.10 | +0.10 | +0.30 | +0.20 | +0.20 | -0.30 | +0.30 | +0.10 |
|  | 25^th^ | +0.70 | +0.30 | +0.50 | +0.60 | +0.40 | -0.10 | +0.40 | +0.50 |
|  | 10^th^ | +0.80 | +0.50 | +0.60 | +0.70 | +0.80 | +0.80 | +1.00 | +1.00 |
|  | 5^th^ | +0.90 | +0.60 | +0.80 | +0.90 | +0.90 | +1.30 | +1.10 | +1.10 |
| 11.5 | 95^th^ | -0.40 | -0.40 | -0.40 | -0.40 | -0.40 | -0.40 | -0.40 | -0.40 |
|  | 90^th^ | -0.40 | -0.40 | -0.20 | -0.40 | -0.40 | -0.40 | -0.30 | -0.40 |
|  | 75^th^ | -0.30 | -0.30 | -0.10 | -0.30 | -0.30 | -0.30 | -0.20 | -0.20 |
|  | 50^th^ | +0.10 | +0.10 | -0.10 | +0.20 | +0.10 | -0.30 | +0.30 | +0.10 |
|  | 25^th^ | +0.60 | +0.30 | +0.10 | +0.60 | +0.40 | -0.10 | +0.40 | +0.50 |
|  | 10^th^ | +0.80 | +0.50 | +0.50 | +0.70 | +0.80 | +0.80 | +1.00 | +1.00 |
|  | 5^th^ | +0.90 | +0.60 | +0.80 | +0.90 | +0.90 | +1.30 | +1.10 | +1.10 |
| 12 | 95^th^ | -0.40 | -0.40 | -0.40 | -0.40 | -0.40 | -0.40 | -0.40 | -0.40 |
|  | 90^th^ | -0.40 | -0.40 | -0.30 | -0.40 | -0.40 | -0.40 | -0.40 | -0.40 |
|  | 75^th^ | -0.30 | -0.30 | -0.10 | -0.30 | -0.30 | -0.30 | -0.20 | -0.20 |
|  | 50^th^ | +0.10 | +0.10 | -0.10 | +0.10 | +0.10 | -0.30 | 0.00 | 0.00 |
|  | 25^th^ | +0.50 | +0.30 | +0.10 | +0.60 | +0.50 | -0.10 | +0.10 | +0.10 |
|  | 10^th^ | +0.80 | +0.50 | +0.50 | +0.70 | +0.90 | +0.80 | +0.20 | +0.40 |
|  | 5^th^ | +0.80 | +0.60 | +0.80 | +0.90 | +0.90 | +1.30 | +0.30 | +0.70 |
| 12.5 | 95^th^ | -0.40 | -0.40 | -0.40 | -0.40 | -0.40 | -0.40 | -0.40 | -0.40 |
|  | 90^th^ | -0.40 | -0.40 | -0.30 | -0.40 | -0.40 | -0.40 | -0.40 | -0.40 |
|  | 75^th^ | -0.30 | -0.30 | -0.10 | -0.30 | -0.30 | -0.30 | -0.20 | -0.20 |
|  | 50^th^ | +0.10 | +0.00 | -0.10 | +0.00 | 0.00 | -0.30 | 0.00 | 0.00 |
|  | 25^th^ | +0.50 | +0.20 | +0.10 | +0.30 | +0.30 | -0.10 | +0.10 | +0.10 |
|  | 10^th^ | +0.60 | +0.50 | +0.50 | +0.60 | +0.40 | +0.80 | +0.20 | +0.40 |
|  | 5^th^ | +0.80 | +0.60 | +0.60 | +0.70 | +0.50 | +1.20 | +0.30 | +0.60 |
